# A Case Report on The Course and Outcome of A Patient Diagnosed with Trichotillomania and Major Depressive Disorder

**DOI:** 10.1192/j.eurpsy.2025.1215

**Published:** 2025-08-26

**Authors:** Z. C. G. Tan, I. C. S. Tan

**Affiliations:** 1 Department of Psychiatry and Behavioral Medicine, University of the Philiippines Philippine General Hospital, Manila, Philippines

## Abstract

**Introduction:**

Trichotillomania (TTM) and Major Depressive Disorder (MDD) are two psychiatric conditions that frequently co-occur, presenting a significant challenge for treatment due to their complex interplay. TTM involves repetitive hair-pulling, leading to noticeable hair loss and distress, while MDD is characterized by persistent low mood and loss of interest or pleasure leading to dysfunctionality.

**Objectives:**

This case report aims to discuss a case of a 21-year-old female with major depressive disorder and trichotillomania, management challenges, and the importance of a comprehensive, multifaceted therapeutic approach to address both disorders effectively.

**Methods:**

A 21-year-old female college student and youth church leader presented with chronic hair-pulling and depressive symptoms. She had low self-esteem and a strong need for validation. Despite her responsibilities, she struggled with emotional distress exacerbated by family dynamics and her church role. Her symptoms were linked to self-esteem threats and feelings of inadequacy. She was diagnosed with Trichotillomania, Scalp, and Major Depressive Disorder.

Initial pharmacologic management was Fluoxetine 20mg/day up titrated to 40mg/day with no improvement hence shifted to Escitalopram 20mg/day and N-acetylcysteine 1200mg/day with noted significant improvement in symptoms. Non-pharmacologic strategies included supportive-expressive psychodynamic psychotherapy, cognitive-behavioral techniques, and family therapy. Psychoeducation, suicide safety planning, and an interprofessional approach with dermatology co-management were also integral.

**Results:**

Over the course of 15 therapy sessions, the patient demonstrated significant improvement in both her depressive symptoms and hair-pulling behavior. Her active engagement in therapy, combined with pharmacological support, facilitated better emotional regulation and a more cohesive sense of self. Her adherence to the treatment plan, along with the collaborative efforts of the interprofessional team, contributed to her positive outcomes.

**Conclusions:**

This case highlights the significance of addressing both TTM and its comorbid conditions for effective treatment outcomes. The interplay between TTM and MDD underscores the need for comprehensive treatment plans incorporating pharmacological and psychotherapeutic approaches. Future practice should consider the benefits of an interprofessional approach for managing complex cases like this.

**Disclosure of Interest:**

None Declared

